# Industrializing traditional medical knowledge in China: an integrated governance framework for addressing institutional fragmentation

**DOI:** 10.3389/fpubh.2026.1889910

**Published:** 2026-08-19

**Authors:** Yanhui Wang, Kai Xu, Dong Hua

**Affiliations:** 1 School of Elderly Care Services and Management, Nanjing University of Chinese Medicine, Nanjing, China; 2 Jiangsu Province Hospital of Chinese Medicine Chongqing Hospital (Chongqing Yongchuan District Hospital of Traditional Chinese Medicine), Chongqing, China; 3 School of Health Economics and Management, Nanjing University of Chinese Medicine, Nanjing, China

**Keywords:** governance fragmentation, intellectual property governance, knowledge governance, regulatory coordination, standardization, traditional medical knowledge (TMK)

## Abstract

**Introduction:**

Traditional medical knowledge (TMK) plays an increasingly important role in contemporary health systems, yet its industrialization remains constrained by fragmented governance across knowledge registration, standardization, and intellectual property (IP) protection. This study examines the institutional barriers affecting TMK industrialization in China within the broader context of public health governance and innovation systems.

**Methods:**

Using qualitative institutional analysis of policy and regulatory documents, this study investigates the institutional arrangements governing TMK registration, standardization, and legal protection, and identifies the mechanisms underlying governance fragmentation.

**Results:**

The study identifies persistent fragmentation across informational, technical, and legal domains that impedes cross-domain institutional coordination. To address these challenges, the article develops an integrated governance framework that aligns TMK registration, standardization mechanisms, and legal protection structures. The findings suggest that this framework improves institutional coordination by clarifying functional linkages among governance domains and reducing systemic misalignment.

**Discussion:**

This study contributes to ongoing discussions on traditional medicine governance and offers policy-relevant insights for improving the coherence and sustainability of health-related knowledge systems This study contributes to ongoing discussions on traditional medicine governance and offers policy-relevant insights for improving the coherence and sustainability of health-related knowledge systems.

## 1 Introduction

The industrialization of traditional knowledge (TK), particularly traditional medical knowledge (TMK), presents a challenge at the intersection of law, economy, and culture. While the transformation of culturally embedded and practice-based knowledge into scalable economic assets enables innovation and market expansion, it simultaneously destabilizes legal frameworks premised on individualized rights and challenges the preservation of TK’s collective and cultural attributes (1).

International governance of TK has been shaped by instruments such as the Convention on Biological Diversity (CBD) (2), which emphasizes conservation, equitable benefit-sharing, and the role of knowledge holders, despite the absence of a universally accepted definition of TK. These challenges are further intensified by the expansion of international trade frameworks, most notably the Regional Comprehensive Economic Partnership (RCEP) (3), which creates new points of interaction between TK governance, intellectual property (IP) regimes, and cross-border commercialization. As TMK continues to expand globally, the need for regulatory coordination across these domains has become increasingly acute.

Despite extensive scholarship, TK governance remains analytically fragmented (4). Existing studies tend to examine IP protection (5), cultural heritage preservation (6), and regulatory frameworks in isolation (7), with limited attention to the institutional interactions that emerge when TK is transformed into industrial and commercial resources. This gap is particularly evident in the context of international trade, where legal, technical, and economic systems converge but remain insufficiently integrated (1).

At the international level, mechanisms such as prior informed consent and access and benefit-sharing (ABS) under the CBD and the Nagoya Protocol continue to face constraints, including fragmented implementation (8), high transaction costs (9), and inconsistencies across domestic legal systems (10). These limitations underscore the difficulty of reconciling global regulatory frameworks with locally embedded knowledge systems (11).

China provides a particularly salient case through which these institutional frictions can be examined. While it has actively promoted the industrialization of TMK through regulatory codification, standardization, and integration into global trade frameworks, its institutional architecture remains fragmented (12). Misalignments between database systems, standardization mechanisms, and IP regimes generate constraints that both reflect and amplify the broader governance challenges surrounding TK (13).

This article argues that the fragmentation observed in TMK governance is not merely institutional, but reflects a deeper structural incompatibility between existing legal regimes and the epistemic characteristics of TMK. Building on this premise, the article reconceptualizes TMK industrialization as a multi-layered governance process structured by the interaction of registration systems, standardization mechanisms, and differentiated legal protection. It demonstrates how fragmentation across these institutional domains produces systemic constraints, including the functional ambiguity of TMK databases, the misalignment between standardization and IP regimes, and insufficient alignment among legal protection mechanisms. In doing so, it situates the Chinese experience within broader global debates and provides analytical insights for the governance of TK-based industries in an increasingly interconnected regulatory environment.

## 2 Dynamic evolution of the industrialization of TMK

### 2.1 Theoretical foundations of the industrialization of TK

The industrialization of TMK can be analytically grounded in two complementary theoretical perspectives, which together provide a robust analytical basis for understanding the transformation of culturally embedded knowledge into industrial assets. First, factor endowment theory (Heckscher–Ohlin framework) highlights the role of natural, human, and institutional resources in shaping regional comparative advantage. Applied to TMK, this perspective suggests that regions with abundant medicinal plant resources, skilled practitioners, and supportive local policies exhibit greater potential for industrial development (14).

Such regional resource and capability configurations explain spatial disparities in TMK industry clusters across China, including the distribution of research, cultivation, manufacturing, and market activities. Factor endowment theory thus provides a macroeconomic rationale for understanding why certain localities are better positioned to industrialize TMK and capture comparative advantage within the global value chain.

Second, knowledge economy and innovation theory complements this resource-based perspective by emphasizing the transformation of tacit, experience-based knowledge into codified, transferable forms as a driver of economic growth (15). TMK represents an experiential and culturally embedded system of understanding (16), and its industrialization regenerates and reutilizes TK resources. Innovation-driven mechanisms enable TMK to evolve from a resource-dependent sector into a knowledge-intensive one, while embedded legal and institutional frameworks—including IP systems, benefit-sharing and cultural heritage protections—shape codification, sharing, and commercialization.

Integrating these perspectives, the industrialization of TMK can be understood as a distinctive process shaped by the interaction of resource endowments, knowledge transformation, and institutional frameworks. While factor endowment theory explains the spatial distribution and material basis of TMK resources, knowledge economy theory highlights the mechanisms through which tacit, experience-based knowledge is codified and rendered transferable. Crucially, this transformation does not occur in a vacuum but is mediated by legal and regulatory institutions that govern access, standardization, and appropriation.

This resource–knowledge–institution nexus distinguishes TMK industrialization from conventional industrial processes. Unlike standard industries, where resources can be directly mobilized for production, TMK requires an additional layer of knowledge articulation and institutional embedding before it can be effectively integrated into industrial systems. This multi-layered interaction underscores the centrality of governance structures in shaping both the opportunities and constraints of TMK industrialization.

### 2.2 The synergistic process of industrializing TMK

**Figure 1:**
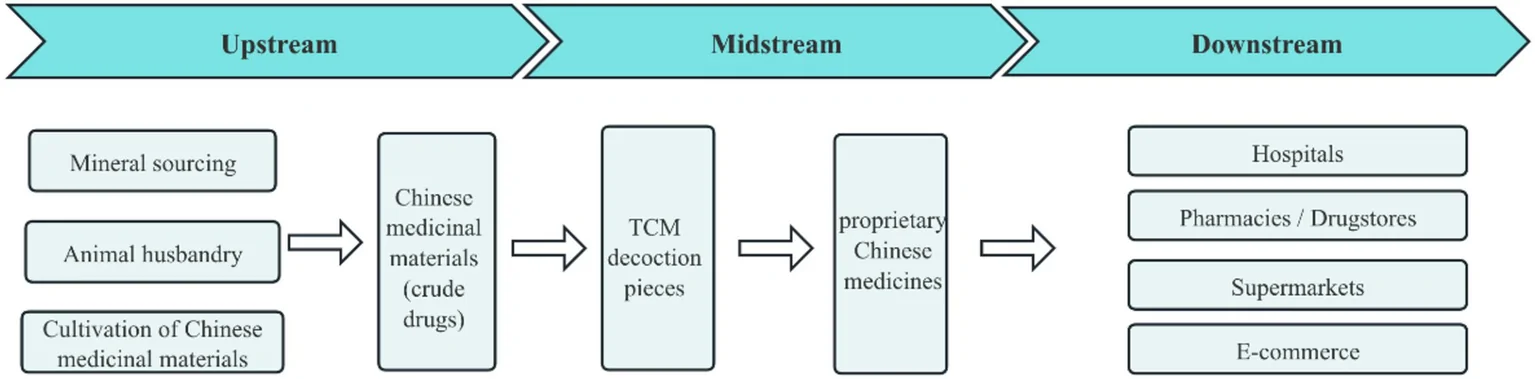
Overview of China’s TMK industrial chain.

At the structural level, China has established a relatively complete TMK industry chain, supported by regulatory frameworks such as the Law of the People’s Republic of China on Traditional Chinese Medicine (TCM) (2017) and related national policy instruments (17). The industry encompasses upstream resource cultivation and research, midstream pharmaceutical manufacturing, and downstream distribution and retail systems (see Figure 1).

On the demand side, rising household income, urbanization, and population aging have significantly increased the demand for TMK-based products. Consumers increasingly seek preventive and wellness-oriented healthcare solutions, while the expanding middle-aged and older population tends to favor TMK due to its perceived holistic benefits, relative affordability, and cultural familiarity (18). These demand-side dynamics not only expand market scale but also interact with regulatory frameworks, influencing classification systems, standard-setting processes, and supervisory mechanisms.

Building on this industrial foundation, the transformation of TMK into scalable economic resources proceeds along two primary dimensions—knowledge codification and technical standardization—supported by legal-institutional alignment. Knowledge codification concerns the transformation of tacit knowledge into explicit forms, while standardization relates to its reproducibility in large-scale production. At the same time, effective industrialization depends on the legal-institutional alignment addressing the cumulative and practice-based characteristics of TMK. Together, these dimensions bridge the gap between localized knowledge systems and industrial application, allowing TMK to operate within modern regulatory and market environments.

The necessity of codification is closely linked to the epistemic characteristics of TMK. Historically, TMK has been embedded in dispersed, practice-based knowledge systems and transmitted through oral traditions (19), limiting its visibility within formal legal and patent frameworks (20). Knowledge confined to individual cognition is inherently vulnerable to loss and cannot easily support industrial-scale utilization. Codification thus serves as a critical interface, rendering knowledge tangible, transferable, and amenable to legal recognition.

In the international context, this function is reflected in initiatives such as India’s Traditional Knowledge Digital Library (TKDL) (21), which has been integrated into patent examination procedures. Such experiences demonstrate that systematic digital codification, as reflected in TK databases, can function not only as a defensive tool against misappropriation but also as an infrastructure enabling the integration of domestic knowledge systems with international IP frameworks (22).

Within this codification pathway, the development of TMK databases has emerged as a key institutional instrument. The RCEP encourages the establishment of TK databases (Chapter 11, Art. 5), positioning them as interfaces between domestic governance systems and international trade and IP frameworks. In China, this process has resulted in the establishment of multiple databases by governmental bodies, research institutions, and enterprises (see Table 1).

**Table 1:** Comparative table of key elements in the operating system for Chinese TCM knowledge databases.

Database	Operating entity	Scope	Intended purpose	Challenges
NMPA Database	National Medical Products Administration (Government Agency)	Public disclosures of filings, including protected TCM varieties, TCM extracts, and TCM formula granules,etc.	Information inquiry/patent searching	Limited international interoperability and mutual recognition
Traditional Chinese Medicine Database System	National Administration of Traditional Chinese Medicine (Government Agency)	Including Chinese medicine journal literature database, disease diagnosis and treatment database, prescription database, ethnic medicine database, etc.	Information inquiry	Limited international interoperability and mutual recognition
TCM Bank	Sun Yat-sen University (Research Institution)	~9,192 herbs, 61,966 ingredients, 15,179 targets, and 32,529 diseases	Information inquiry	Insufficient updates and maintenance
World Traditional Medicine Patent Database (WTMPD)	Beijing East Linden Science and Technology Co., Ltd. (Enterprise)	~700,000 traditional-medicine patents worldwide and 250,000 TCM prescriptions	Patent searching	Insufficient updates and maintenance

These databases facilitate the aggregation, classification, and dissemination of TMK-related information, improving knowledge accessibility and reducing information asymmetries across research, regulatory, and industrial domains (23). By enabling cross-referencing of heterogeneous data and lowering search and transaction costs, they enhance the efficiency and scalability of TMK utilization, while also revealing the growing complexity of multi-agency governance (24).

Parallel to codification, the industrialization of TMK relies on the advancement of standardization. The standardization of TCM involves the formulation and implementation of technical standards to ensure the safety, efficacy, stability, and consistency of products (25). Beyond its technical function, standardization operates as a core institutional mechanism for enhancing consumer trust and facilitating market expansion in both domestic and international contexts (26). At the same time, the increasing incorporation of proprietary technologies into technical standards introduces potential institutional frictions between public regulatory objectives and private IP rights, foreshadowing the institutional challenges discussed below.

In the context of globalization, such standards also function as key technical factors shaping international trade (27). China has actively promoted the standardization of TCM since the mid-2000s, seeking to establish internationally recognized quality standards and regulatory norms (28). This strategy not only supports large-scale industrial production but also links domestic regulatory systems with global trade and IP frameworks (29).

However, despite the progressive advancement of codification and standardization, their legal-institutional alignment remains incomplete. These governance challenges are further reflected in the industrial application of TMK, where different technological methodologies interact with regulatory and standardization frameworks in different ways. Representative TMK-related formulations, including TCM formula granules and TCM varieties, have already achieved industrial-scale implementation through mature pharmaceutical manufacturing methodologies. Before turning to the broader governance analysis, Table 2 provides an overview of major TMK-related methodologies currently applied in industrial practice and the principal institutional constraints associated with their implementation.

**Table 2:** Representative TMK-related methodologies in industrial practice and their institutional challenges.

Process category	Representative TMK-related technologies in industrial application	Current industrial status	Major institutional challenges
Extraction	Supercritical fluid extraction; macroporous adsorption resin purification; membrane separation (ultrafiltration/nanofiltration)	Mature and established in large-scale pharmaceutical production	Technical standardization of scale-up processes; regulatory requirements for process modifications; harmonization with international quality standards
Granulation	Fluidized-bed granulation; dry granulation (roller compaction)	Mature and applied in GMP-certified facilities for formula granules and oral dosage forms	Regulatory requirements for process modifications and continuing challenges in harmonizing TCM-specific formulation standards.
Quality control	Chromatographic fingerprinting (HPLC/GC); near-infrared (NIR)-based process analytical technology (NIR-PAT)	Chromatographic fingerprinting is mature and codified; NIR-PAT remains in transition from pilot applications to broader industrial deployment	Limited regulatory acceptance of real-time PAT data; further harmonization is needed across analytical platforms and pharmacopoeial standards

As Table 2 illustrates, TMK industrialization extends beyond the commercialization of products and involves differentiated institutional arrangements across technological methodologies. Representative methodologies have reached different stages of industrial-scale implementation, ranging from mature and codified applications to technologies that remain in transition toward broader industrial deployment. Extraction, granulation, and quality-control methodologies are associated with distinct technical, regulatory, and standardization challenges, reflecting varying degrees of technological maturity and institutional readiness. Recognizing these differences is essential for understanding how technological development and institutional design interact during the industrialization process. The following section therefore examines the broader institutional challenges of TMK industrialization from the perspectives of knowledge codification, technical standardization, and legal protection.

## 3 Analysis of the constraints in the industrialization process of TMK

### 3.1 Functional deviation of TMK databases

From the perspective of knowledge economics, establishing a TK database supplemented by rules of prior informed consent, disclosure of origin, and benefit sharing under the CBD, represents a key institutional response for the effective governance of TK (30). The mechanism addresses the “free-rider” problem arising from the non-rivalrous nature of knowledge (31), enabling greater traceability and facilitating its potential tradability through codification. Ultimately, it ensures that creators and custodians of TK receive appropriate incentives and rewards (32). This may include both economic incentives and recognition mechanisms, providing motivation for holders to maintain, document, and share TK systematically.

Within this framework, user countries can employ such databases as platforms for negotiation with source countries, thereby reducing the transaction costs associated with accessing TK resources. Furthermore, by integrating patent search and examination interfaces, these databases can link domestic patent systems to the international application mechanism under the Patent Cooperation Treaty (PCT), thereby facilitating patent examination and prior art verification.

However, the functional orientation of these databases remains ambiguous (33), as it is unclear whether their primary objective is to promote benefit sharing, prevent the misappropriation of TMK in patent applications, or affirm rights over TK. To date, no consensus has been reached among database developers and governmental authorities on this issue. In practice, their potential remains underutilized due to the absence of a clearly defined functional orientation (34).

In the absence of specific and operable legislative guidance, Chinese research institutions, enterprises, and local governments have independently developed databases with inconsistent standards and incompatible interfaces, leading to information fragmentation, which may reduce data reliability and constrain industrial integration (24). As a result, many existing databases lack authority, and data collection often follows no objective criteria, exhibiting considerable arbitrariness (35).

Furthermore, the relationship between TMK databases and the IP system remains ambiguous. Most databases currently operate merely as information-display platforms, lacking formal linkage with international patent examination procedures and systematic integration with global databases managed by WIPO or WHO. Consequently, they cannot provide legally recognized evidence for the utilization of TMK at the international level. Functionally, these databases primarily serve internal research and regulatory purposes rather than effectively supporting the broader development of the TMK industry (24).

From a regulatory perspective, the digitalization of TMK, if confined to mere disclosure and retrieval functions, is insufficient to ensure effective protection or rational utilization. As argued by Dutfield and Suthersanen (36), integrating TK into modern IP frameworks that mandate compulsory disclosure creates a fundamental tension.

While undifferentiated disclosure increases the risk of unauthorized access to confidential or undisclosed knowledge (i.e., technical secrets) (37), it discourages knowledge holders from disclosing such knowledge, thereby impeding the systematic transformation of tacit knowledge into explicit, manageable, and industrially applicable forms.

### 3.2 Bottlenecks in the standardization of TCM

Driven by market incentives, the industrialization of TMK increasingly depends on the effective standardization of TCM. Since technical standards in TCM often involve technological solutions and methods of implementation—elements frequently protected under IP regimes—there exists an inherent interrelationship between standardization and IP rights (38).

**Figure 2:**
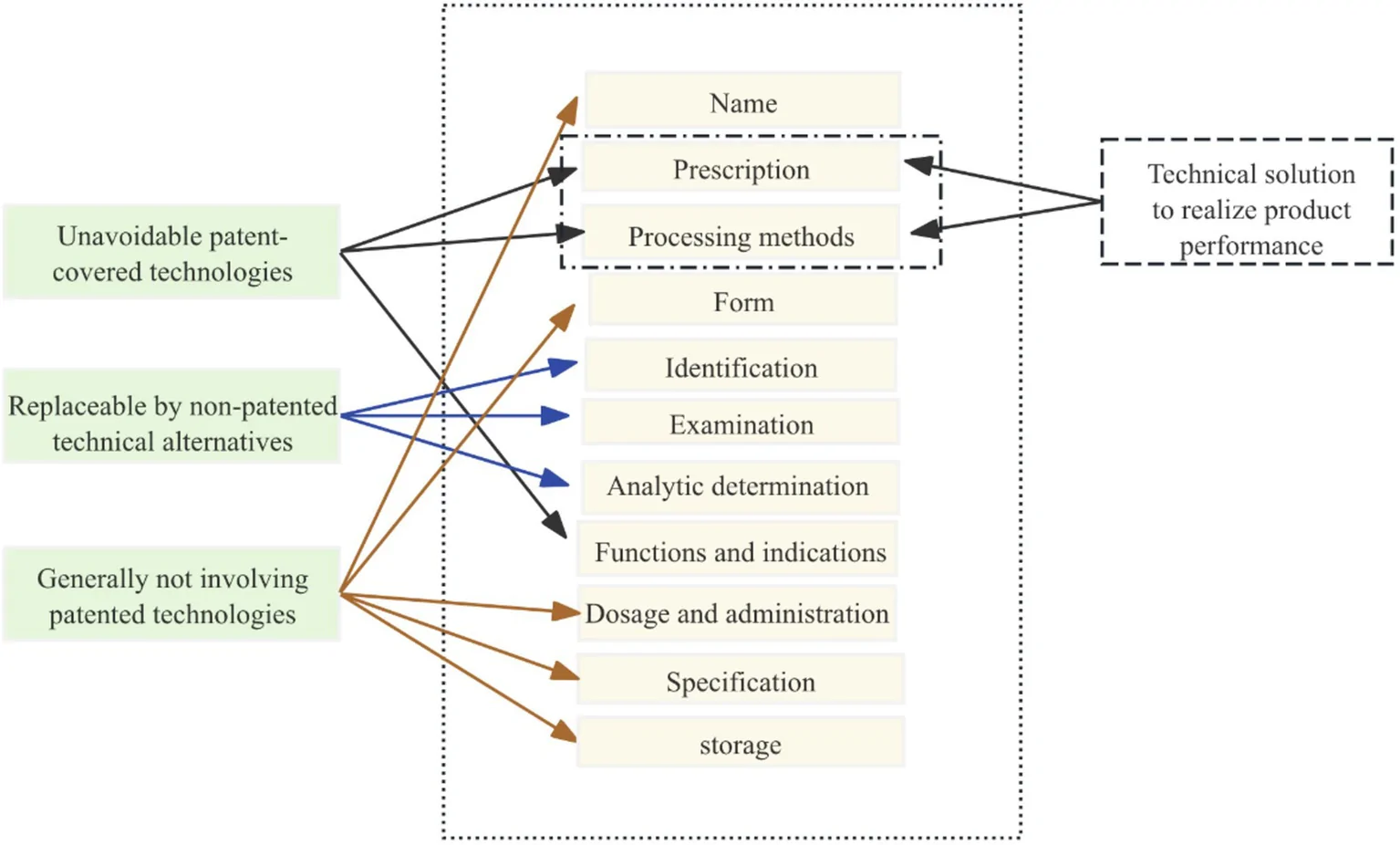
Schematic diagram of the correlation between TCM standards and TCM patents.

IP rights related to TCM standards are primarily patent-based (see Figure 2), and may also encompass technical secrets. While these rights operate within the domain of private law, technical standards function as public regulatory instruments that articulate objective technical norms. The overlap between private IP rights and public standardization creates legal and operational complexity, as discussed below.

This dilemma is particularly pronounced in the field of standardization (39). On the one hand, to ensure the quality and efficacy of TCM, technical standards must emphasize its distinctive, experience-based processing and preparation methods, which stand in contrast to the performance-oriented indicators typically applied in chemical drug regulation. On the other hand, when such traditional processes are appropriated as exclusive rights through patent protection and subsequently incorporated into mandatory standards, a structural incompatibility emerges between the public nature of TK and the exclusivity inherent in IP regimes.

This type of tension, widely discussed in the literature on standard-essential patents and innovation governance (40), is increasingly reflected in the context of TCM standardization (41).

At the practical level, this misalignment can delay the adoption of standardized practices, increase legal uncertainty for manufacturers, and generate uneven competitive conditions across the industry. Smaller enterprises, in particular, often lack the institutional and financial capacity to navigate the interaction between patent constraints and mandatory standards, thereby exacerbating asymmetries within the market.

While the formulation of such standards is formally a deliberate act by standard-setting bodies, their actual market influence is largely shaped by commercial dynamics and firm adoption behavior (42). In practice, market forces often play a decisive role in determining which patented technologies become widely adopted in industrial settings, thereby influencing the allocation of research and development resources within enterprises.

In this context, the integration of patented technologies into TCM standards can, to some extent, provide a viable pathway for addressing conflicts between standards and IP rights. However, an assessment of patented technologies included in current TCM standards suggests a relatively low level of inventiveness in many cases. Many of these patents reflect incremental modifications and high homogeneity rather than substantial, differentiated innovations, which limits their capacity to add significant clinical or industrial value (43).

Consequently, the foundational patents underpinning these standards often fail to withstand market scrutiny during their validity period. Furthermore, many standards continue to rely on conventional industrial techniques with limited process controllability (44), which not only affects the reproducibility and consistency of products but also further constrains the international recognition and competitiveness of both TCM patents and standards.

### 3.3 Misalignment among legal protection mechanisms

Once incorporated into the legal protection framework, TMK has transitioned from a master–apprentice transmission model to a legally recognized form of “property,” acquiring the potential to be evaluated and traded in the market. From a legal perspective, the protection of TMK involves the intervention and maintenance of rights. In modern contexts, the industrialization of TMK makes the choice of appropriate protection mechanisms crucial from the outset of industrial development. Effective institutional design is therefore essential to ensure that knowledge protection aligns with both domestic industrial objectives and international legal norms.

If the documentation and digitalization of TMK enhance its identifiability and communicability, and the standardization of TCM improves its applicability and replicability, a robust legal safeguard framework is essential to facilitate the valorization of such knowledge and support the sustainability of its industrial application. Within this framework, the IP system—particularly the patent regime—plays a pivotal role. In line with global trends emphasizing information disclosure and knowledge sharing, the patent system has been widely recognized for promoting technological diffusion and stimulating pharmaceutical innovation (45).

Nevertheless, as a product of modern legal institutions, the patent system often proves inadequate in protecting traditional forms of knowledge such as TCM. Its formal requirements for subject-matter eligibility, novelty, and inventive step fail to accommodate the experiential, cumulative, and context-specific nature of TMK, leaving many valuable formulations insufficiently protected. In some cases, patents diverge from the theoretical foundations of TCM by focusing primarily on the qualitative and quantitative analysis of individual components, making it difficult to fully capture or effectively regulate their clinical safety and efficacy. Consequently, the patenting of TCM faces considerable challenges, with both grant rates and the scope of protection falling short of expectations (43).

In response to these limitations, Chinese regulatory authorities have explored supplementary mechanisms beyond the patent system. The TCM Varietal Protection System (TCMVPS) has emerged as an alternative form of protection (46). Compared with patent protection, the TCMVPS adopts a more flexible threshold, placing less emphasis on formal novelty and instead prioritizing practical utility in determining eligibility and protection levels. This functional orientation reflects the distinctive characteristics of TMK, which is often cumulative, experience-based, and transmitted across generations rather than arising from discrete inventive steps. By focusing on therapeutic efficacy and established usage, the TCMVPS provides a more inclusive legal pathway that accommodates the incremental and collective nature of TMK, thereby facilitating its contribution to public health and industrial development (47).

Institutionally, the TCMVPS establishes a dual-tier protection structure (48). Class I protection operates in a manner analogous to trade secret protection, under which key technical information—including formulations and manufacturing processes—remains undisclosed during the protection period. In contrast, Class II protection functions primarily as a regulatory exclusivity mechanism, allowing only a limited number of authorized enterprises to produce the protected variety subjects to administrative approval and supervision. This differentiated structure reflects a hybrid regulatory logic that combines elements of IP protection with administrative control over market entry.

Since its introduction over three decades ago, the TCMVPS has played a significant role in advancing TMK industry, fostering the development of numerous innovative pharmaceutical products (49). Nevertheless, as a system unique to China and heavily reliant on administrative protection, the TCMVPS has yet to achieve effective coordination with the patent regime, revealing a misalignment between domestic institutional design and international IP norms.

**Figure 3:**
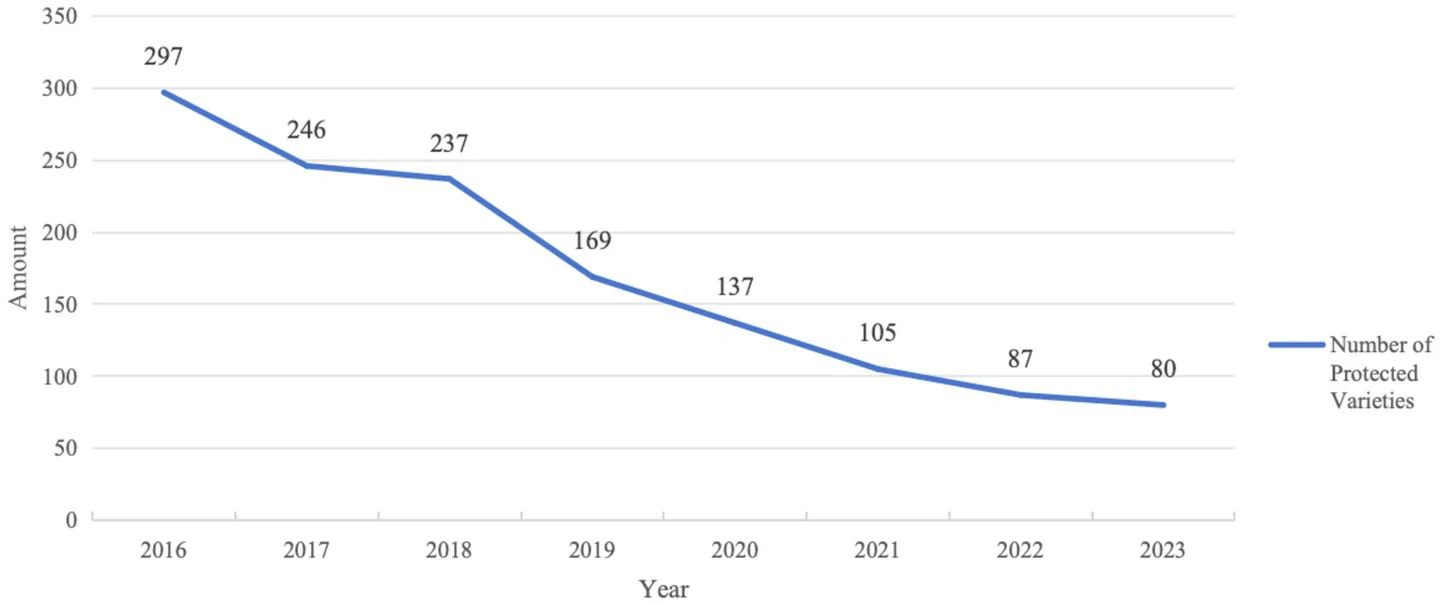
Trend in the number of protected TCM varieties (2016–2023). Source: author’s compilation based on official TCMVPS announcements published on the NMPA website.

This misalignment can create uncertainty for innovators, limit cross-border commercialization opportunities, and complicate compliance with global IP standards. As a result, the future trajectory of the system remains uncertain, with the number of varieties under protection showing a gradual decline as of 2023 (see Figure 3). This situation underscores the need for institutional harmonization to support both innovation and compliance with global IP standards.

## 4 Toward integrated pathways for TMK industrialization

Building on the preceding institutional analysis, this section advances an integrated governance framework that synthesizes registration systems, standardization mechanisms, and differentiated legal protection into a coherent analytical model. Rather than re-specifying individual protection instruments, the focus here is on how these elements interact as an interacting system to enable the scalable integration of TMK into innovation and regulatory systems.

At its core, the framework consists of three integrated components.

First, a registration layer establishes traceability and attribution by systematically recording TMK in differentiated forms, distinguishing between publicly accessible knowledge and confidential technical know-how. This layer provides a foundational infrastructure for clarifying entitlements, enabling prior art verification, and supporting benefit-sharing arrangements.

Second, a standardization interface translates registered knowledge into technically applicable and reproducible forms, facilitating its incorporation into industrial production and regulatory systems. By aligning technical standards with knowledge characteristics, this layer ensures consistency, quality control, and scalability across the value chain.

Third, a differentiated legal protection layer allocates exclusive or semi-exclusive rights across different forms of TMK, building on the functional distinctions between mechanisms such as patents, technical secrets, and administrative protections discussed earlier.

### 4.1 Enhancing the digital registration system for TMK

Within the broader context of industrialization, the digitalization of TMK represents both a result of its modernization and a strategic response to the evolving dynamics of international biotrade. Art. 53 of Chapter 11 of RCEP (3) introduces specific provisions regarding databases, encouraging member states to promote the circulation, sharing, and equitable benefit-sharing of TK. This provision highlights that the challenge for member states is no longer simply the creation of TK databases, but their effective design, governance, and operational integration into both domestic and international knowledge ecosystems (37). Moreover, it implies the need for interoperability standards and data governance protocols to ensure that knowledge can flow seamlessly across borders while respecting national sovereignty and cultural heritage rights.

Accordingly, the registration of prior rights in TMK should extend beyond merely preventing improper patent applications or grants. A core function of TMK databases should aim to clarify the scope of rights associated with recorded knowledge and to facilitate its authorized use and industrial application. In addition, clear registration practices can serve as a reference for dispute resolution, providing evidence in legal or commercial conflicts involving knowledge misappropriation. Differentiating between publicly disclosed TK and undisclosed technical secrets during registration strengthens legal certainty and provides a practical foundation for robust digital governance.

Comparative insights can be drawn from international experiences. India’s TKDL systematically digitizes Ayurvedic, Unani, and Siddha medicinal knowledge and integrates it into patent examination processes, thereby functioning as a legal and institutional safeguard against misappropriation, facilitating patent examination and reinforcing domestic IP enforcement. By codifying knowledge in standardized digital formats and linking it with patent offices, the TKDL model exemplifies how digital registration and legal recognition can jointly protect TK while supporting industrialization (50).

In 2021, the Chinese National Administration of TCM (NATCM) issued the Draft Regulations on the Protection of TMK (for Public Consultation) (51), marking the first step toward establishing a formal registration procedure. This initiative not only formalizes the legal recognition of TMK as an industrial asset but also signals China’s intent to harmonize domestic digital governance with emerging international standards, reflecting an awareness of cross-border technology transfer and IP protection issues. Implementing such a system represents both a prerequisite for coherent database creation and a necessary condition for realizing systematic digital management and traceability of TMK.

Under the current design, TMK databases classify entries according to transmission status, target users, degree of disclosure, and confidentiality requirements. For non-public knowledge, registration is voluntary and initiated by the knowledge holders. Such classification also facilitates differentiated access rights, allowing regulatory authorities to monitor compliance while supporting commercial use under controlled conditions. This design not only enables clear attribution of rights to data but also underscores the need for sufficient legal incentives and risk-prevention mechanisms to encourage active participation by knowledge holders.

When selecting incentive measures, policy stability and sustainability should be prioritized. Direct government rewards or financial subsidies for knowledge registrants are difficult to institutionalize, particularly in the absence of reliable criteria for evaluating the industrial application of specific TMK. Furthermore, excessive reliance on direct subsidies could distort market incentives, leading to inefficiencies in knowledge deployment and potential overvaluation of low-impact entries. Without such evaluative standards, authorities face challenges in determining equitable compensation or assessing the potential impact of knowledge on industrial development. By contrast, a market-based mechanism—where knowledge users negotiate directly with holders—offers a potentially more practical approach. Under this model, non-public databases may operate on a fee-based basis, serving primarily as intermediaries that organize and process knowledge information, while users provide consideration to access and utilize it. This arrangement reduces search and transaction costs, encourages efficient allocation of resources, and promotes innovation by linking knowledge use directly to economic incentives (52).

From the perspective of promoting innovation, confirming ownership of undisclosed TMK constitutes an incentive for holders. Although non-public databases focus on collecting non-disclosed information, they actively encourage knowledge flow. These platforms allow knowledge holders to license or assign their technical secrets, generating revenue from transactions and optimizing the deployment of TK resources. This “benefit-return” mechanism accelerates the commercialization of TMK by motivating holders to leverage their information in accordance with cost–benefit considerations. Moreover, it establishes legitimate channels for controlled dissemination and application of non-public knowledge, preventing inefficient hoarding and encouraging broader industrial use.

### 4.2 Harmonizing TCM standardization and IP strategies

WHO has elevated the international recognition and development of traditional medicine to a key global health priority, as enshrined in its newly adopted Global Traditional Medicine Strategy 2025–2034 (53). This strategy systematically charts a course for the next decade, aiming to strengthen the evidence base—for instance, through international clinical trial registration—while also promoting the application and dissemination of knowledge.

Nevertheless, a significant challenge persists: the legal frameworks for protecting TK remain predominantly national or regional, resulting in widely varying quality standards that reflect disparate local public health needs and healthcare systems. Coordinating these legislative differences across countries is essential to ensure the safety, efficacy, and credibility of traded knowledge or derivative products, and it also requires capacity-building efforts, policy harmonization, and enhanced cross-border cooperation among regulatory bodies, posing a substantial challenge for both WHO and TK source countries.

As a normative system encompassing both technical and managerial aspects, the standardization of TCM faces multiple practical challenges. In addition to the absence of comprehensive quality evaluation methods and the limited specificity of quality control indicators, legal constraints require alignment between TCM standards and the IP system, particularly patents. Accordingly, alignment across technological, legal, and industrial dimensions becomes essential to address the misalignments between standardization and IP regimes. Such alignment ensures compatibility between technical standards and IP rights, while facilitating the transformation of TK into industrially applicable forms.

Notably, Japan’s experience in standardizing Kampo medicine offers a valuable benchmark. In this context, comparative experiences provide useful insights into how such alignment can be institutionally achieved. Over several decades, Japan has harmonized clinical, pharmacopoeia standards, and manufacturing standards for Kampo formulations, ensuring product consistency, quality, and regulatory compliance across both domestic and international markets (54).

This process of institutional alignment has been largely government-led, with the Ministry of Health, Labor and Welfare and related regulatory agencies playing a central role in standard-setting and quality regulation (55, 56). Pharmacopoeial standards and manufacturing requirements have been progressively harmonized, ensuring that Kampo formulations are produced according to unified specifications under the Japanese Pharmacopoeia and GMP frameworks.

Compared with innovation models that rely more heavily on proprietary claims and patent protection, the Japanese system places greater emphasis on regulatory standardization and quality control, highlighting the role of institutional alignment in ensuring product safety and consistency. As a result, this institutional alignment reduces fragmentation between technical standards and regulatory frameworks, providing a stable basis for large-scale industrial production.

This case illustrates how coordinated standardization and regulatory integration can support scalable production while preserving the therapeutic and cultural integrity of traditional knowledge. It also reflects the importance of aligning resources, knowledge, and institutional frameworks, as discussed earlier. For China’s TMK industry, the Japanese example provides a practical reference for integrating complex TMK formulations into standardized, market-ready products without compromising either clinical efficacy or cultural significance.

TCM standardization thus reflects the need for a holistic framework that extends across the entire production-to-market chain, reflecting the complexity and multi-stage transformation of TMK into industrial products. This entails the development of a comprehensive quality evaluation system encompassing herbal materials, decoction pieces, extracts, and proprietary Chinese medicines, as well as the establishment of operational quality control indicators for critical technical processes, including extraction, purification, and formulation. Such an integrated approach is essential to ensure both scientific rigor and practical feasibility in the standardization process.

Specifically, efforts can be made in the following three aspects. At the technical level, integration between TCM standardization and international resource chains should be strengthened. As a source country of TK, China has taken a leading role in this effort, as evidenced by the work of the International Organization for Standardization (ISO)‘s TCM Technical Committee (ISO/TC 249), which is led by China. By the end of 2023, ISO/TC 249 had published 95 international standards for TCM and is currently developing 31 additional standards (57).

However, the current international standardization efforts largely focus on single-herb materials, leaving polyherbal formulations and clinical practice standards relatively underrepresented. The limited scope of current standards creates challenges for industrial translation, as most TMK practices involve complex interactions of multiple herbs, compounded processing techniques, and individualized prescriptions. Direct evidence of this trend is found in the Committee’s issuance of Technical Report ISO/TR 23975:2025, TCM—Priority List for Developing International Standards on Single-Herb Materials (58). The report explicitly confines its scope to establishing prioritization frameworks for international standardization of single-herb materials. Such a narrow scope underscores the necessity of developing a more inclusive standardization strategy that captures the full spectrum of TMK and clinical practice.

At the legal and institutional level, a coordinated mechanism between TCM standards and patents should be established. This entails considering existing patent rights during standard-setting to avoid exclusive conflicts, clarifying the legal status of technical solutions incorporated into standards, safeguarding public usability while protecting IP, and developing incentive policies that encourage enterprises to engage in standard formulation, technological innovation, and application. Furthermore, these mechanisms should include guidance on harmonizing domestic regulations with international IP frameworks, facilitating global market access while maintaining domestic innovation incentives. Additionally, formal dispute resolution mechanisms are necessary to mitigate legal risks and provide clarity on IP-standard interactions.

At the industrial level, insufficient engagement by enterprise actors can exacerbate the disconnect between TCM standards and market demand. To address this, standardization should be closely integrated with enterprise research and development, production, and market access, ensuring that standardized outcomes inform industrial strategies. Dynamic monitoring systems and periodic review mechanisms are also necessary to ensure that standards evolve in line with emerging technologies, new clinical evidence, and shifting consumer preferences. A dynamic updating mechanism should be implemented to maintain the relevance of standards in response to technological advancements and evolving market conditions.

Furthermore, international coordination can be strengthened by encouraging the formation of cross-border enterprise standard alliances within the TMK sector. By creating platforms for cross-border collaboration, knowledge exchange, and joint innovation, enterprises can better leverage global IP protections and strengthen the international adoption of TCM standards. By strategically leveraging IP protections to promote technological innovation, these measures can enhance the global recognition and adoption of TCM standards.

It is worth noting that, in the above respects, some Chinese pharmaceutical enterprises have already made relatively successful attempts. A representative example is Shanghai Xingling Pharmaceutical and its ginkgo leaf extract products (59). As the marketing authorization holder, the company actively aligned TCM patent development with standard-setting. While filing patents for ginkgo leaf extract and its preparation methods, Xingling established internal enterprise control standards in the early 2000s and subsequently contributed to the drafting of national pharmacopoeia standards (60). Through this process, the company’s research outcomes were transformed into public standards, converting patent achievements into industry norms and establishing a sector-level technological leadership position. At the same time, product standards were harmonized with European and U. S. pharmacopoeias, promoting the internationalization of ginkgo preparation quality standards.

This case illustrates how strategic alignment of IP and standards can simultaneously drive industrial innovation, market competitiveness, and regulatory harmonization. This “patent–standard–market” linkage mechanism not only enhanced the practical value of Xingling’s IP but also exemplifies an innovative development model in TCM, where standardization supports industrial competitiveness and IP guides technological standardization.

### 4.3 Functional differentiation between the TCMVPS and patent system

Within the legal framework for TMK protection, stakeholders across different sectors are incentivized to assert and negotiate private rights. While fully self-enforcing rights align with private law principles, their practical implementation is often hindered by institutional, informational, and procedural challenges. A foundational approach that combines private law protections with collaborative safeguards—such as specialized rights, registries, and benefit-sharing mechanisms—provides a conceptual basis for addressing these challenges (61).

Such collaborative safeguards may include administrative oversight, coordinated standard-setting, and registry mechanisms that collectively reduce the risk of disputes and clarify entitlements for all parties. The core issue lies in effectively integrating existing legal frameworks to maximize their protective functions, which should guide both system design and practical application.

In the context of TMK, one concrete institutional form of such collaborative safeguards is the TCMVPS. The establishment of the TCMVPS was initially intended to incentivize innovation and facilitate the industrial application of TCM, particularly for medicines for which patent protection is insufficient (62, 63). Consequently, improving the legal protection framework for TMK requires a clear functional delineation between supplementary systems such as the TCMVPS and the patent regime. Rather than operating as overlapping mechanisms, these systems should be structured as complementary instruments addressing different categories of knowledge.

Specifically, the functional differentiation between the TCMVPS and the patent system should not be understood as a matter of policy preference, but as an institutional response to the epistemic heterogeneity of TMK. Knowledge that is cumulative, experience-based, and often partially undisclosed does not readily conform to the formal requirements of patent law, particularly with respect to novelty, inventiveness, and disclosure. In such cases, the TCMVPS provides a more adaptable protective framework by accommodating evidentiary flexibility and emphasizing demonstrated therapeutic value. By contrast, where TMK-derived innovations exhibit clearly identifiable technical features—such as novel compounds, extraction techniques, or specific therapeutic applications—they become compatible with the epistemic and procedural structure of patent law and are more effectively governed within that regime.

This differentiation thus reflects an alignment between forms of knowledge and modes of legal protection, in which each system operates according to its comparative institutional capacity to capture, evaluate, and regulate distinct epistemic attributes.

The need for transitional mechanisms between the TCMVPS and the patent system arises from the dynamic and evolving nature of TMK itself. As knowledge moves from tacit, experience-based forms toward more codified and technically defined expressions, its compatibility with different legal regimes may also shift over time. A rigid allocation of protection mechanisms is therefore insufficient to accommodate this epistemic fluidity. In this context, institutional linkages—such as phased protection structures, conditional conversion pathways, and coordinated licensing arrangements—can be understood as adaptive responses that enable the reconfiguration of rights in accordance with changes in the form and evidentiary status of knowledge. Rather than merely serving as policy instruments, these mechanisms function to bridge otherwise discontinuous legal regimes, allowing knowledge to transition between different modes of protection without generating regulatory gaps or overlaps.

By facilitating this continuity, such mechanisms mitigate the fragmentation identified earlier and enhance legal predictability, while preserving the flexibility necessary to govern heterogeneous and evolving forms of TMK.

## 5 Conclusions and future directions

This study demonstrates that the industrialization of TMK is not merely a process of resource utilization, but a structured transformation shaped by the interaction of resources, knowledge, and institutions. This perspective highlights the distinctive nature of TMK industrialization, which requires not only the mobilization of material resources but also the articulation of experiential knowledge and its embedding within regulatory and legal frameworks.

To support such industrialization, an integrated legal protection framework for TMK offers three normative advantages. First, it enhances legal certainty by clarifying the status and function of TMK across different regulatory domains; second, it improves institutional efficiency by reducing duplication and inconsistency between databases, standards, and IP regimes; third, it strengthens innovation incentives while preserving the cultural and collective dimensions of TMK, by enabling flexible and context-sensitive rights allocation.

These components can be understood as interacting through a dynamic governance process: registration enhances information transparency and reduces asymmetries, thereby enabling both standard-setting processes and IP claims to operate on a more reliable evidentiary basis; standardization stabilizes knowledge inputs and reduces uncertainty in industrial application, facilitating the effective deployment of IP rights; meanwhile, the allocation of IP rights generates economic incentives that encourage participation in registration systems and the disclosure—or controlled sharing—of knowledge.

Through this interaction, the framework aligns informational, technical, and legal dimensions of TMK governance, mitigating the fragmentation observed in existing institutional arrangements. By embedding TMK governance within a layered and coordinated institutional architecture, this framework provides a structured pathway for reconciling the competing objectives of knowledge preservation, commercial utilization, and regulatory coherence. Although grounded in the Chinese context, it offers broader analytical value for other jurisdictions seeking to integrate TK into contemporary IP and innovation systems.

Building on this analytical premise, the study examines three interrelated dimensions of TMK governance: the design of knowledge codification (through digital registration systems), technical standardization, and the alignment of legal protection mechanisms. Together, these elements reveal how preconditions for industrialization, domestic regulatory dynamics, and external governance structures interact to shape both opportunities and constraints.

China’s legal and regulatory approach to the industrial development of TMK should therefore be guided by the recognition of this complexity. Aligning domestic regulatory practices with international frameworks not only enhances participation in cross-border trade but also contributes to safeguarding the intellectual and cultural dimensions of TK. More broadly, an integrated governance approach offers a coherent pathway for balancing commercialization with heritage preservation, thereby supporting the sustainable development of TK-based industries.

Several limitations should be acknowledged. First, the analysis primarily draws on legal texts and institutional frameworks, with limited attention to on-the-ground implementation or industrial practice. Second, comparative exploration of TK protection in other states remains underdeveloped. Future research should further explore the cross-border industrialization of TMK, focusing on the challenges at the intersection of legal protection, industrial development, and international governance. Key issues include the design and enforcement of IP frameworks, particularly regarding patenting, ownership attribution, and protection of communal rights; the harmonization of technical and quality standards to facilitate international trade without compromising the integrity of TK; the interoperability of TMK registries and TK databases, which is essential for prior informed consent, benefit-sharing, and effective information exchange; and international trade barriers, including tariff and non-tariff regulations, that influence the global circulation of TMK products. Comparative analyses of cross-national governance experiences could provide additional insights, informing policies that promote the sustainable industrialization of TMK while preserving its cultural, ethical, and communal dimensions.

By situating TMK at the intersection of cultural heritage, IP, and international trade, this study highlights the governance challenges arising from the industrialization of TK in a globalized regulatory environment. It further demonstrates the importance of institutional coordination across registration systems, standardization mechanisms, and legal protection frameworks. Ultimately, the findings suggest that a multi-layered governance strategy combining digital registration, harmonized standards, and differentiated legal protections may provide a comparative framework for TK-holding countries seeking to balance knowledge protection, industrial development, and regulatory coherence within the global knowledge economy.

## Data Availability

The original contributions presented in the study are included in the article/supplementary material, further inquiries can be directed to the corresponding authors.

## References

[1] Qin TB Dong JY. Benefit orientation and risk avoidance: legal protection for the industrialization of traditional knowledge. Academic Research. (2020) 7:59–66.

[2] Convention on Biological Diversity (CBD) (1992) Text of the convention. Available online at: https://www.cbd.int/convention/text/. Accessed 24 May 2026.

[3] RCEP (2024) Regional comprehensive economic partnership agreement: Full Text. Available online at: https://asean.org/wp-content/uploads/2024/10/Regional-Comprehensive-Economic-Partnership-RCEP-Agreement-Full-Text.pdf. Accessed 24 May 2026.

[4] Milchior R. WIPO treaty on IP, genetic resources and associated traditional knowledge: a new international agreement, but does it really create a full new IP right? J Intellect Prop Law Pract. (2025) 20:129–30. doi: 10.1093/jiplp/jpae081, 40388063

[5] Robinson DF Abdel-Latif A Roffe P, editors. Protecting Traditional Knowledge: The WIPO Intergovernmental Committee on Intellectual Property and Genetic Resources, Traditional Knowledge and Folklore. London: Routledge (2017).

[6] Srinivas KR. Protecting traditional knowledge holders’ interests and preventing misappropriation—traditional knowledge commons and biocultural protocols: necessary but not sufficient? Int J Cult Prop. (2012) 19:401–23. doi: 10.1017/S0940739112000252

[7] Hua H Tang JY Zhao JN Wang T Zhang JH Yu JY . From traditional medicine to modern medicine: the importance of TCM regulatory science (TCMRS) as an emerging discipline. Chin Med. (2025) 20:92. doi: 10.1186/s13020-025-01152-8, 40563101 PMC12199503

[8] Richerzhagen C. The Nagoya protocol: fragmentation or consolidation? Resources. (2014) 3:135–51. doi: 10.3390/resources3010135

[9] Nakanyete NF Matengu KK Revilla Diez J. Rich resources from poor communities: an analysis of Namibia's access and benefit-sharing legislation. Environ Dev. (2024) 49:100943. doi: 10.1016/j.envdev.2023.100943

[10] Schebesta H. The potential of private standards for valorizing compliance with access and benefit sharing obligations of genetic resources and traditional knowledge. Agronomy. (2021) 11:1823. doi: 10.3390/agronomy11091823

[11] Avilés-Polanco G Jefferson DJ Almendarez-Hernández MA Beltrán-Morales LF. Factors that explain the utilization of the Nagoya protocol framework for access and benefit sharing. Sustainability. (2019) 11:5550. doi: 10.3390/su11205550

[12] Hu WJ Hu W Li M Chi X Wang X Khan AU. Intangible cultural heritage research in China from the perspective of intellectual property rights based on bibliometrics and knowledge mapping. Hum Soc Sci Commun. (2024) 11:825. doi: 10.1057/s41599-024-03314-9

[13] Xia N. Intellectual property protection for traditional medical knowledge in China’s context: a round peg in a square hole? Med Law Rev. (2023) 31:358–90. doi: 10.1093/medlaw/fwad006, 37018625 PMC10452053

[14] Zhu X Zhang Z Xu L Zhong X. Current situation and countermeasures of traditional Chinese medicine resource distribution: a case study of Wuyi County in China. Front Public Health. (2025) 13:1473833. doi: 10.3389/fpubh.2025.1473833, 40313505 PMC12043565

[15] Choong KK Leung PW. A critical review of the precursors of the knowledge economy and their contemporary research: implications for the computerized new economy. J Knowl Econ. (2022) 13:1573–610. doi: 10.1007/s13132-021-00734-9, 40477203 PMC7995391

[16] Lyu S Zhao Z Liu G Zhou S. Understanding embodied experiences in a traditional Chinese medicine-based health promotion program: insights from in-depth interviews and participant observations. Health Commun. (2026) 41:52–62. doi: 10.1080/10410236.2025.2490230, 40256996

[17] State Council Information Office of China (2016) Traditional Chinese Medicine in China Available online at: https://english.www.gov.cn/archive/white_paper/2016/12/06/content_281475509333700.html. Accessed 24 May 2026.

[18] China Press of Traditional Chinese Medicine (2022) Blue book highlights growth of traditional Chinese medicine industry. Report summary. Available online at: https://govt.chinadaily.com.cn/s/202303/23/WS6422a54a498ea274927b34be/blue-book-highlights-growth-of-traditional-chinese-medicine-industry.html. Accessed 24 May 2026.

[19] Adekannbi J. Olatokun W. Ajiferuke I. (2014) Preserving traditional medical knowledge through modes of transmission: a post positivist enquiry, S Afr J Inf Manag 16:#598. doi: 10.4102/sajim.v16i1.598

[20] Wang YH Xu K. Related issues and responses in the process of industrialization of traditional knowledge of Chinese medicine. Chin Pharm. (2025) 36:140–5. doi: 10.6039/j.issn.1001-0408.2025.02.02

[21] Fredriksson M. India’s traditional knowledge digital library and the politics of patent classifications. Law Crit. (2023) 34:1–19. doi: 10.1007/s10978-021-09299-7, 36915708 PMC9999701

[22] Hilty RM Batista PH Carls S. "Traditional knowledge, databases and prior art: options for an effective defensive use of TK against undue patent granting". In: Stamatoudi I, editor. Research Handbook on Intellectual Property and Cultural Heritage. Cheltenham: Edward Elgar Publishing (2022)

[23] Zhang YQ Li X Shi Y Chen T Xu Z Wang P . ETCM v2.0: an update with comprehensive resource and rich annotations for traditional Chinese medicine. Acta Pharm Sin B. (2023) 13:2559–71. doi: 10.1016/j.apsb.2023.03.012, 37425046 PMC10326295

[24] Wang YY Liu M Jafari M Tang J. A critical assessment of traditional Chinese medicine databases as a source for drug discovery. Front Pharmacol. (2024) 15:1303693. doi: 10.3389/fphar.2024.1303693, 38738181 PMC11082401

[25] Leong F Hua X Wang M Chen T Song Y Tu P . Quality standard of traditional Chinese medicines: comparison between European pharmacopoeia and Chinese pharmacopoeia and recent advances. Chin Med. (2020) 15:76. doi: 10.1186/s13020-020-00357-3, 32742301 PMC7388521

[26] Guo DA Guo D Wu W Yao C Da J. Comprehensive analysis and elaboration of quality standard for traditional Chinese medicines. Planta Med. (2021) 87:1238. doi: 10.1055/s-0041-1736741

[27] Haider R. International trade in pharmaceutical products. Int J Appl Econ Account Manag. (2023) 1:227–50. doi: 10.59890/ijaeam.v1i4.913

[28] Wang J Guo Y Li GL. Current status of standardization of traditional Chinese medicine in China. Evid Based Complement Alternat Med. (2016) 2016:9123103. doi: 10.1155/2016/9123103, 27110268 PMC4826698

[29] State Council of China (2024) Action Plan for Standardization of Traditional Chinese Medicine (2024–2026) Available online at: https://www.gov.cn/zhengce/zhengceku/202407/content_6965475.htm. Accessed 24 May 2026.

[30] Tang XD Bian Q. The cognition of standards and standardization concepts and reflection on standardization work of traditional Chinese medicine specialty. Guidel Stand Chin Med. (2024) 2:2–6. doi: 10.1097/gscm.0000000000000015

[31] Dasgupta P David PA. Toward a new economics of science. Res Policy. (1994) 23:487–521. doi: 10.1016/0048-7333(94)01002-1

[32] Foray D. The Economics of Knowledge. Cambridge, MA: MIT Press (2004).

[33] Jacoby CD Weiss C. Recognizing property rights in traditional biocultural contribution. Stanf Environ Law J. (1997) 16:74–124.

[34] Hardison P (2005) The report on traditional knowledge registers (TKRs) and related traditional knowledge databases (TKDBs), UNEP/CBD/WG8J/4/INF/9, prepared for the Secretariat of the Convention on Biological Diversity Available online at: https://www.cbd.int/doc/meetings/tk/wg8j-04/information/wg8j-04-inf-09-en.pdf. Accessed 24 May 2026.

[35] Zhang LX Dong J Wei H Shi SH Lu AP Deng GM . TCMSID: a simplified integrated database for drug discovery from traditional Chinese medicine. J Cheminform. (2022) 14:89. doi: 10.1186/s13321-022-00670-z, 36587232 PMC9805110

[36] Dutfield G Suthersanen U. Dutfield and Suthersanen on Global Intellectual Property law. 2nd ed. Cheltenham, UK; Northampton, MA: Edward Elgar Publishing (2020).

[37] Li YD. On the trends of India’s traditional knowledge database management system and the enlightenment. Intellect Prop. (2020) 3:86–96.

[38] Vásquez Callo Müller M Ortega Sanabria DF Matsuno Remigio A. The WIPO treaty on intellectual property, genetic resources and associated traditional knowledge: situating a landmark development in international intellectual property governance. GRUR Int. (2024) 73:1128–36. doi: 10.1093/grurint/ikae140

[39] Gervais DJ. Traditional knowledge and intellectual property: a TRIPS-compatible approach. Mich St L Rev. (2005) 1:137–94.

[40] Hall BH Helmers C. "Technology standards and standard essential patents". In: Hall BH Rosenberg N, editors. The Economics of Innovation and Intellectual Property. Oxford: Oxford University Press (2024). p. 511–34.

[41] Xu Y Wang YM. "IP protection of traditional knowledge: a dilemma faced by the Chinese traditional medicine in global competition". In: Prabu SL Umamaheswari A, editors. Intellectual Property: Global Perspective Advances and Challenges. London: IntechOpen (2023)

[42] van de Kaa G. Standards adoption: a comprehensive multidisciplinary review. Heliyon. (2023) 9:e19203. doi: 10.1016/j.heliyon.2023.e19203, 37664752 PMC10468385

[43] Li X. Influencing factors, evolution, and strategies for improving the quality of traditional Chinese medicine patents: an empirical study based on patent invalidation cases. Ann Med. (2024) 56:2422571. doi: 10.1080/07853890.2024.2422571, 39484709 PMC11536629

[44] Zhang JW. Collaborative Development between Traditional Chinese medicine Standardization and Intellectual Property Strategy. Beijing: Intellectual Property Publishing House (2011).

[45] Dutfield G. Intellectual Property rights and the life Science Industries: a Twentieth Century History. London: Taylor & Francis (2017).

[46] Jiang GN . Research on the traditional Chinese medicine varietal protection system. Leg Syst Econ. (2020) 10:94–5.

[47] Li WL (2025) Evolution of the protection system for traditional Chinese medicine varieties and the strategic responses of traditional Chinese medicine enterprises Available online at: https://journal.hep.com.cn/ajtm/EN/1188148588674420765. Accessed 24 May 2026.

[48] State Council of China (1992) Regulations on the protection of traditional Chinese medicine varieties. Available online at: https://www.wipo.int/wipolex/en/text/337300. Accessed 24 May 2026.

[49] He Q Ma SC. Review of the 30 years since the implementation of the regulations on the protection of traditional Chinese medicine varieties. Chin Pharm J. (2023) 24:2209–12. doi: 10.11669/cpj.2023.24.002

[50] Council of Scientific and Industrial Research of India (CSIR) (n.d.) The Traditional Knowledge digital Library unit (TKDL) Available online at: https://www.csir.res.in/en/documents/tkdl. Accessed 24 May 2026

[51] NATCM (2021) Draft regulations on the protection of traditional Chinese medicine knowledge (for Public Consultation) Available online at: https://www.natcm.gov.cn/fajiansi/gongzuodongtai/2021-10-25/22994.html. Accessed 24 May 2026.

[52] Li YD. On explanation, analysis and countermeasures for the article of traditional knowledge database in RCEP. Intellect Prop. (2022) 6:92–109.

[53] WHO (2025) Global Traditional Medicine Strategy 2025–2034 Available online at: https://www.who.int/westernpacific/publications/i/item/9789240113176. Accessed 24 May 2026.

[54] Arai I Kawahara N. Kampo pharmaceutical products in the Japanese health-care system: legal status and quality assurance. Trad Kampo Med. (2018) 5:35–44. doi: 10.1002/tkm2.1204

[55] Goda Y. Regulatory science of natural products. J Nat Med. (2022) 76:732–47. doi: 10.1007/s11418-022-01639-w, 35870047 PMC9307968

[56] Maegawa H Moore K. Enhancing value of global pharmacopoeia standards: summary of joint USP–MHLW/PMDA workshop. BPB Reports. (2025) 8:50–63. doi: 10.1248/bpbreports.8.2_50

[57] China Daily (2023) New TCM Institute in Shanghai unveiled. Available online at: https://www.chinadaily.com.cn/a/202306/05/WS647d7c0da31033ad3f7ba859.html. Accessed 24 May 2026

[58] ISO (2025) Traditional Chinese Medicine — Priority List for Developing International Standards on Single-Herb Materials, Technical Report ISO/TR 23975:2025 Available online at: https://www.iso.org/standard/86397.html. Accessed 24 May 2026.

[59] Shanghai Pharmaceuticals Xingling Sci. & Tech. Pharmaceutical Co., Ltd. (n.d.) Company profile Available online at: https://www.shlys.com/channels/24.html. Accessed 24 May 2026.

[60] SPH Xing Ling Sci. & Tech. Pharmaceutical Co., Ltd PatSnap (n.d.) Company profile and patent portfolio Available online at: https://discovery.patsnap.com/company/sph-xing-ling-scitech-pharmaceutical/. Accessed 24 May 2026

[61] Wright E. Protecting Traditional Knowledge: Lessons from Global case Studies. Cheltenham, UK: Edward Elgar Publishing (2020).

[62] Cai TQ Song XT. Necessity and rationality of exclusive protection of the traditional Chinese medicine varieties. Chin Pharm J. (2023) 58:2217–20. doi: 10.11669/cpj.2023.24.004

[63] Diligena D Song XT. Current situation of protection system for varieties of traditional Chinese medicines. Chin Herb Med. (2022) 53:3870–80.

